# Letter from Dr. Harlan—No. VI

**Published:** 1839-09-28

**Authors:** 


### MEDICAL EXAMINER.

DEVOTED TO MEDICINE, SURGERY, AND THE COLLATERAL SCIENCES.

No. 39.] PHILADELPHIA, SATURDAY, SEPTEMBER 28, 1839. [Vol. II.

### FOREIGN CORRESPONDENCE.

### LETTER FROM DR. HARLAN—No. VI.

*Want of Good Faith among French Physicians—Indian Anecdote—M. Magendie's Treatment of Neuralgia by Electricity—M. Serres' Memoir on the Functions of the Respiratory Bronchial Apparatus in the Embryo—M. Mirbel on Calorification in Living Vegetables—Baron Larrey on the employment of Gold Leaf in Small Pox—Visit from Baron Larrey—M. Guérin's Memoir on Sub-cutaneous Wounds—M. Velpeau's Operation for Calculus without dividing the neck of the Bladder—M. Velpeau's Failure with Jacobson's Cassepierre—Treatment of Colica Pictonum at La Charité—M. Bouillaud and his Amphitheatre—Lecture and Operations by M. Civiale—The Salpétrière—"Affiches" of Paris—Royal Veterinary School and Hospital at Alfort—Description of the Jardin des Plantes—Liberality of the Professors to Dr. H., on the loss of his Museum—Baron Duportet's Lectures on Animal Magnetism—List of Gratuitous Lectures delivered at Paris.*

Paris, August 10, 1839.

To the Editors of the Medical Examiner.

An American Physician, devoted to his profession, and acclimated for a few months in Paris, would find no difficulty in writing volumes, derived from his daily observations. The *materiel* exists in the greatest profusion, but much of it is of a quality which renders it useless, and unworthy of attention—to separate truth from humbug, the chaff from the wheat, is the great difficulty. Generally speaking, the character of the medical profession has here reached its last degree of deterioration; and this just appreciation of the public is mainly due to the shocking disregard of truth which is displayed in the verbal and written statements of medical men. There is no knowing when and what to believe: during their most grave polemical discussions, both in and out of the "Royal Academy of Medicine," they not unfrequently give the unsophisticated lie to each other, without the least hesitation. In private practice, it is not uncommon for them to abuse, underrate, and under-bid each other; and they have consequently, with the loss of self-respect, lost the regard and confidence of the public. Is there not a fearful approach to this much to be reprobated state of the profession, in many of the Atlantic cities of the United States? and is it not a shame that man, in the highest state of civilization, should sacrifice every noble and honourable feeling, and merge the high behests of science, in self-interest and love of "filthy lucre?" As a commentary, I will here mention an instance of savage virtue that occurred among our aborigines of the far west, during the last war between Great Britain and the United States:—"A beaver-trapper returning from an excursion to the countries bordering the Rocky Mountains, halted at the lodge of an old friend, the chief of a tribe of Indians inhabiting the Upper Missouri. During the evening, the voice of the news-crier was heard announcing the arrival of an Indian traveller from the "Great Lakes," then the principal seat of belligerent operations. The chief proposed to his guest to go along with him to meet the principal men of the village at the traveller's lodge, when, after a few moments of characteristic and becoming silence of the party, the Indian traveller commenced his history, and gave an animated and interesting detail of the action between the opposing fleets, in which the Anglo-Americans proved victorious. Not a word of comment was made by his austere audience, who separated in silence. The white man expressed his surprise to his Indian host at the apparent apathy of his race, who could listen to such a glowing account with so little interest;—the host rejoined, that his people experienced no want of interest, but remarked emphatically of the traveller, "*That man once told a lie!* What he has now said, may be true—may be false."

A distinguished London physician, eminent for his experimental investigations in physiology, once remarked to me, that he had long ceased even to read any memoirs published by the profession in Paris,—asserting that he had always found the truth that they might contain so enveloped in a mass of verbiage, and every degree of misstatement, from the simple poetical gloss to unsophisticated falsehood, that he was utterly unable to derive any benefit from their labours. There are some honourable exceptions to this general implication, from whose friendly intercourse I have derived both pleasure and advantage. A professional resident in Paris possesses great advantages in this respect, being enabled, by his own observations, to verify the numerous statements and discoveries which are daily made public. Availing myself of this privilege, I shall now proceed to note, for the benefit of such of my compatriots as interest themselves in your journal, such events as appear to me most worthy of notice, occurring since the date of my last letter. I commence with the transactions at the séances of the Academy of Sciences. This department of the National Institute of France is unquestionably one of the gravest and most dignified assemblages in the world, consisting, with few exceptions, of men of every variety of profession, ardently devoted to the interests of science, and sincerely in search of truth and knowledge.

*Séance commencing 17th June*, 1839.—M. Magendie stated his success in the treatment of neuralgia, and of paralysis of the facial nerve, by the application of electricity directly to the nerves affected, by means of platina needles; he gave the detail of the treatment of two cases, in which the deformity arising from the paralysis of the muscles of the face was very great. The pain, in the first case, was driven, by repeated applications of the needle, from one nerve to the other, and finally altogether expelled. Mr. M. offered to introduce me to these patients, if I was disposed to question them on their disease and treatment. M. Serres presented at the same séance, a memoir "On the respiratory bronchial apparatus of the human embryo, during the earlier months of its development." He supposes that the membranes perform the function of lungs, at this period of gestation. Haller carried this notion still further in his day, asserting that in the membrane of the egg of the chicken, the animal itself *pre-existed.* Neither of these authors have, in my opinion, illustrated satisfactorily their position, by the facts which they have brought to bear upon it.

M. Mirbel lately presented a memoir detailing certain careful experiments, which appeared to prove satisfactorily the existence, in living vegetables, (in the stems of trees and shrubs, for example,) of a power to generate heat. The stalks of plants and trees, it appears, possess a proper heat only during their soft or herbaceous state. M. M. could never trace the least degree of this proper heat in the *ligneous* tissue of trees, notwithstanding his thermo-electric apparatus could readily detect the existence of one-sixty-fourth of a degree of centigrade of heat, corresponding to one-fourth of a degree of deviation of the magnetic needle of the multiplicator. This experiment must be made within doors, in a room with a northern exposure, in order to obviate the effects of solar reflection.

Last year, the Baron Larrey communicated a note to the Academy, announcing that the Egyptians and the Arabians are accustomed to preserve the faces of young persons from the ravages of the small pox, by covering them with gold leaf at the moment of the invasion of this disease. M. Legrand communicated to the Academy, at a late séance, the result of a recent experiment of his, with this method, on a young English lady of great beauty, afflicted with confluent small pox. From the first appearance of the eruption, to the termination of the suppurative fever, he covered the whole surface of the face with gold leaf, night and morning,—the leaf being prepared like that used for the cold gilding, and which he caused to adhere to the skin by the use of gum-water. With the exception of some spots on the side of the face, where the gilding was disturbed by the friction of the pillow, the face, although it had experienced great tumefaction, was perfectly preserved from cicatrix, and the features had resumed all their delicacy: the hands, which had not been subjected to the same process, were marked with the usual scars. M. Larrey remarked that, in Egypt, they gilded the hands and feet also, the latter being habitually naked. M. L. also laid on the table, for the inspection of the members, the foot of a female mummy, on which the gilding, or gold leaf, was still apparent; but, notwithstanding Baron Larrey's conviction, this was not positive proof that the gilding on this foot had been intended to answer a similar purpose. It is highly gratifying to see this respectable veteran in science still enjoying a green old age, and as busily as ever interesting himself in the pursuits of knowledge. The flattering honours conferred upon this Nestor of army surgeons, by Napoleon, and many of the crowned heads of Europe, would have made avaricious of praise many a sterner front than his; and, hence, vanity, although a principal feature, is yet a venial flaw in his character: the polite attentions which he is so ready to yield to the claims of others, render the Baron an agreeable companion. He honoured me with a visit a few evenings since, in company with his son, already a distinguished member of the profession. In the course of conversation, he alluded to his early visit to North America, having made his first campaign during the revolution. This fact had escaped my memory at the time, and, on my expressing some surprise, he turned towards his son with a look of despair, and said, "He has then not read my military campaigns." I assured him of his mistake, but that nearly twenty years had elapsed since I had that gratification. He was appeased, and excused my oblivious reminiscence.

M. Guérin, whose orthopedic institution, near Passy, I alluded to in my last, has just finished reading an interesting memoir before the Academy of Sciences, entitled "Memoire sur les plaies sous-cutanées," in which he has announced a real and useful surgical discovery. I dwell more at large on this subject, as Dr. Mott informs me that he has witnessed the success of this operation by Dr. G., whose reputation stands deservedly high. Dr. G. having observed the frequent absence of all inflammatory symptoms after the operation for dividing the tendo Achillis, when this simple operation was skilfully performed, extended his views to other tissues, and other parts of the body; and he has successfully proved that when such sub-cutaneous wounds are attended by inflammation, it is entirely due to the communication of the external air with the interior of the wound. He made, in fact, about two hundred sub-cutaneous sections of tendons in different parts of the body, by means of a simple puncture of the skin; and in no case was the operation followed by inflammatory reaction. He next proceeded to determine if all the other tissues of the body could be divided, and all sub-cutaneous wounds, of however great extent, would be followed by similar results. These questions were to be solved by experiments both on man and animals. He first divided transversely the longitudinal vertebral muscles in dogs: the experiment was many times repeated in different portions of the same muscles, by means of a simple puncture of the skin, and the cure always took place without inflammation in twenty-four or forty-eight hours. He subsequently made a large incision beneath the skin, from the root of the neck to the sacrum, dividing all the fleshy mass lying along the spine. On the same animal, the whole fleshy mass of the posterior and superior part of the thigh, from the bone to the skin, was divided transversely, taking special care to make a very small opening in the skin, and immediately to close this opening after the experiment, by means of a single suture. In every case, there occurred considerable effusion in the intervals of the divided tissues, and beneath the skin; but this effusion was always absorbed in twenty-four or forty-eight hours, and the cure was effected without inflammation, by means of a peculiar intervening organized material, (au moyen d'un travail d'organisation immédiate,) without the least appearance of fever, the animals continuing their functions nearly as in a natural state. There was to be observed on a level with the wounds, an accumulation of a soft, gelatinous matter, which gradually acquired the consistence of the other tissues. The dogs in which the fleshy mass of the thighs had been divided, were at first completely paralysed; but this affection progressively diminished, and disappeared almost completely in ten or twelve days.

Passing from animals to men, M. G. divided successively beneath the skin, at first for the treatment of chronic torticollis, the tendons and muscles of the neck, the sterno-cleido-mastoideus, trapezius, and the angularis scapulæ; afterwards, for the treatment of the lateral curvatures of the spine, he made the section of the great muscles of the back and column, the common masses of the sacro-lumbar and the long dorsal, the transverse spinal, &c., comprising the aponeuroses, the tendinous sheaths, the vessels and nerves which occupy or traverse these tissues. In all these operations, the wounds presented phenomena analogous to those which had been observed in animals; and in every case, save one, the wounds became immediately organized, without the least trace of inflammation. In the single case which did not follow the regular progress of the others, there resulted a little inflammation and suppuration; but, in this instance, two openings had been made in the skin, the openings were larger than ordinary, and a certain quantity of air had been introduced into the interior of the wound, finding its way between the cellular sheaths of the long dorsal muscles. After this accident, greater care was taken to make only a simple puncture in the skin, and to expel, after each operation, any air that might have been introduced into the wound, and to close the little opening with diachylon plaster, when the immediate organization of the wounds followed, without the least appearance of local or general reaction.*

I shall not occupy your attention any further, at present, with my gleanings from the séances

* See an article by M. Guérin, on "Lateral Deviations of the Spine," No. 37, p. 594.

of the Academy of Sciences. Varied and extensive as are the subjects discussed at the weekly reunions of this learned body, the student in every department of the physical sciences will find here gratification and profit. I have listened with great pleasure to the instructive memoirs on comparative anatomy of Professors Blainville and Duvernoi,—the former treating principally of fossil osteology, the latter of the structure and functions of the organs of respiration in the mamminiféres and fishes, the organs of lactation, &c., of the former. Both these distinguished professors are actively and successfully occupied in enlarging the boundaries of this most important department of science.

Since the death of Dupuytren, the Hôtel Dieu has lost the almost exclusive attraction for the surgical student which it once possessed; La Charité, St. Louis, La Pitié, with the Hôpital des Véneriens, almost equally divide his attention. For historical as well as operative surgery, Velpeau, at La Charité, is undoubtedly a favourite. The female patient operated on by M. V. for calculus, without dividing the neck of the bladder, mentioned in my last communication, was discharged cured during the latter part of last month, without difficulty of retention of urine. If this is to be a general result of this method of operating, it will constitute a great improvement.

M. V. operated a few days since for stone in the bladder; the instrument used was a modification of Jacobson's cassepierre; the patient was a healthy young man, but by no means a favourable case for this method of operating, the stone being too large and too hard. But M. V. was induced to try it from his desire to show this method of operating to his class; he not only failed in seizing and breaking the stone, but brought away with the instrument, shreds of the mucous coat of the bladder. The patient fell a victim to this display of theatrical surgery, dying in a few days of inflammation of the bladder, and irritative fever. I have observed some cases of colica pictonum treated in Fouquier's ward, at La Charité; Rochelle salts, emetic tartar, and injections, are the principal means employed; opiates are resorted to only late in the disease, on the seventh or eighth day; some practitioners use croton oil. The results of French practice in this disease are not very successful.

Bouillaud, also, lectures on pathology in this hospital. His amphitheatre is the best I have seen in any of the French hospitals, only it is too much surrounded by houses and trees: the ceiling is lofty, and decorated with a fresco painting, an allegory, intended to represent Hippocrates on on a cloud, supported and led by Minerva. The walls above the heads of the pupils display inscriptions calculated to impress them with elevated notions of their profession, viz., opposite the Professor,—"Notre esprit est comme une terre, et les leçons des maitres, sont comme la semence qu'on y jette."—*Hippoc.*

"Pour étudier la médicine avec succes, il faut des dispositions naturelles—des connaissances, un lieu favorable, une education premiére, l'amour du travail, du tems."—*Hippoc.*

Behind the Professor,—"Si la santé est le plus précieux de tous les biens, et qu'il n'y est pas de bonhèur sans elle, conserver la vie et la santé, doit être la plus belle de toutes les sciences et la plus recommandable pour tous les hommes."—*Hoffman.*

"Ne faites jamais quelque chose d'important d'après une pure hypothese ou une opinion."—*Max. Stoll.*

On the Professor's right hand,—"J. N. Corvisart, né le 15 Février, 1755, mort le 18 Septembre, 1821. Institution de la chaire de Clinique interne,—Maladies du Cœur,—Percussion."

On the Professor's left hand,—"R. T. H. Laennec, né le 17 Février, 1781, mort le 13 Aout, 1826. Anatomie Pathologique, Auscultation."

Over the large door of the amphitheatre, outside,—"Ecole Clinique ouverte au Vij., Profess. J. N. Corvisart."

M. Bouillaud is a man of spare habit, about forty years of age, good cerebral developments, and has distinguished himself by his researches in pathological anatomy. His recently published work on the diseases of the heart, proves him to be a worthy occupant of the chair of his distinguished predecessor, Corvisart. M. B.'s lecture this morning was on the general clinique of his wards; his *élèves* listened to him with attention.

In order to witness the "beau ideal" of surgical operations, one must frequent the "Hôpital Neckar," the theatre of Civiale's fame. The "explorations" and operative manipulations of one of the most eminent and experienced of surgeons, in that delicate and important class of disorders which occupy his chief attention, constitute a model for the imitation of all who aspire to similar success. His operations have been very frequent during the last two months, and resulted in his usual happy manner. He has been frustrated occasionally by the very great size and hardness of the stone, in which case he generally hands over his patient to Bèrard, who performs the high operation. This is the case with an elderly patient now under treatment, in whose bladder there is a calculus two and a half inches in diameter, and which is too hard to be crushed by the cassepierre, and too large to be grappled by the arms of the lithontripteur.

In a recent lecture, M. C. remarked that the size, length, and curvature of the urethra vary in different subjects; the curvature being modified by the size and extent of the *triangular ligament*; this latter being also modified by the crura of the pubis: hence no set form of curvature of the catheter could be equally applicable to all urethræ. In exploring with his lithontripteur, which is nearly straight, except at its inferior extremity, there is occasionally a difficulty in passing the triangular ligament and neck of the bladder, in which case he depresses the proximate extremity of the instrument, almost in a line with the axis of the patient's body, and keeps up a constant and continued, but gentle pressure in this direction for several minutes, which generally succeeds in accomplishing the passage of the instrument. Previously to operating, he elevates the hips of the patient with a sheet folded into a pad of two feet square, or yet more elevated; the urine is then drawn off with a silver cathether, and five or six ounces of cold water are injected; the catheter is withdrawn, and the proper instrument introduced, which is generally accomplished with so much skill, and ease to the patient, that the instrument appears to glide in of its own accord. When fairly entered, the beak of the cassepierre is turned towards the rectum; the stone, if of the proper size and consistence, is seized and crushed by the pressure of the palm of the hand; if it resist this, the circular screw, or percussion, is resorted to.

During the preparative treatment of the patient, in dilating the urethra, or in accustoming the parts to the presence of instruments, he is careful not to allow the patient to discharge all his urine, in order to avoid the pressure of the bladder against the stone, which would cause great irritation.

In going the rounds of the hospitals of this great metropolis, the foreign student of medicine should not neglect to make a visit or two to the "Salpétrière," one of the largest institutions of the kind in the world, devoted exclusively to females, poor, aged, infirm, idiots, and maniacs. The extensive buildings of this establishment were originally destined for a saltpetre manufactory; hence the name of the hospital. The necessary alterations were made, and additional buildings constructed, to fit it for its present use, many years ago. At present, six thousand inmates are comfortably accommodated, and yet not all the beds are occupied; fifteen hundred of these are idiots, and four hundred others occupy the sick wards. I met Dr. Falret here, by appointment, some weeks ago; he is one of the chief physicians, and invited me into his cabinet, containing some hundred plaster busts of the most extraordinary malformations of the cranium which have occurred during many years in this vast magazine of ugliness, where the "divine image" will be observed to have been subjected to every species of deformity, or conceivable alterations of the skull, that the wildest visions of phrenology could have hoped for. My attention was next directed to numerous cases, both in the the wards and at large, of "*tribades*" and "*nymphomania*," with all their disgusting concomitants of vice and disease, including females of every age,—some whose heads had been bleached by fifty winters, in whom the fires of lust continued to burn with unabated vigour,—figuratively represented by Mount Etna, the snow on whose top has failed to quench the burning lava beneath. I was glad to make my escape from one of these hoary amazons, who made demonstrations not to be mistaken, to the apparent edification and delight of her disgusting comrades. In many cases, where epilepsy, fatuity, &c., are induced by such unnatural aberrations, the professional attendants have resorted to instruments, devised expressly to prevent the manual interference of these patients. That such degrading and ruinous propensities are by no means confined to the wretched inhabitants of the Salpétrière, is sufficiently illustrated by certain "affiches," which for years have occupied the windows in the Palais Royal, the fashionable resort of both sexes, from all countries.

But in Paris the eye of the stranger is met at every glance with shocking proofs of the depravity, and the total absence of delicacy and refinement which characterize the busy multitude which crowds the streets in every direction; the walls are covered with indecent inscriptions and "affiches." Fine oil paintings are hung up over the doors in all quarters of the city, representing a splendidly dressed female, decorated with bonnet and feathers, a full-length portrait, carrying a red-faced infant—"nouveau-né"—in her arms, with the appropriate inscription, "Madame ——, Sage femme." Passing up that populous street, Rue St. Dennis, near the Boulevard, lately, my attention was drawn to the sign of "a regular trader," of enlarged views in these matters. An immense tableau occupied a conspicuous and elevated front of a house, representing a family picture, the figures as large as life; there were the "lit d'accouchement," "une accouchée," "une accoucheuse," bearing a ptisane, "une nourrice," holding the nouveau-né, and "une amie de famille," leaning over the patient, inquiring after her health. Not far from this "modest production," on the Boulevard itself, may be seen decorating "une maison d'accouchement," in a recess over the door, a wax figure of a lady, as large as life, lying upon *straw!* These are but a few instances of the less gross obscenities which offend the eye of uninitiated modesty throughout this metropolis of "the politest nation of the earth!" But what better could be anticipated from the government of a "Most Christian Majesty," in which *religion* constitutes no part, and whose renegade subjects have either altogether banished the *Sabbath* from their calendar, or use it only for the grossest and noisiest exhibitions, as if in ridicule of what they appear to recognise only as a former prejudice. I am sure that if a pious Methodist parson would take a promenade on a Sunday evening along the Champs Elysées, it would kill or cure him! It is time to quit this episode, and return to our walk round the wards of the Salpétrière. I joined Dr. Pariset, who follows Dr. Falret, from 9 to 10 o'clock, A. M., three times a week. Of the two or three hundred patients he actually prescribed for at this visit, there were very few of any practical interest to the student, consisting principally of the infirmities of age, or cases of chronic mental affections. The considerate kindness of the prescribing physician—the attention and care of the numerous nurses, particularly of the Sisters of Charity, whom the patients appeared to worship—the prevalent order and cleanliness, are the most remarkable characteristics which strike a stranger on his first visit to the wards of this vast establishment. As ordinary diet, broth and potage take the place of bread and milk in our hospitals. There are two large bathing houses, placed conveniently to the wards, each adapted to accommodate twelve patients; they were at present all occupied with patients, chattering at each other all at once, like magpies—their bodies being all modestly concealed by a horizontal wooden cover, with an opening to accommodate the neck, and of a size to prevent the possibility of drowning either by accident or design, when the patients are left for half an hour soaking by themselves. It has been ascertained, that in France there exist more female than male idiots,—the reverse of what occurs in England, and perhaps also in the United States. Some of the numerous inclosures contain small brick houses and gardens, where patients of better circumstances, and who can afford to pay a trifle, receive extra attentions.

I subsequently made a visit to "Bicétre," two miles south of the walls of Paris. This is a similar institution to the Salpétrière,—only it is devoted exclusively to the male sex,—among whom, if idiotism is less frequent, the demoniacs are proportionably more numerous. Dr. *Ferrus*, second only to the venerable *Esquirol* of the Charenton hospital, for the treatment of the insane, delivers an interesting series of lectures here on Sunday mornings, from 9 to 10 o'clock. Notwithstanding the inconvenient distance of this hospital from Paris, Dr. F. is followed by a select and attentive audience, among whom I recognised Dr. Lajus and Dr. Spencer, of Philadelphia.

It is hardly possible to conceive that a truly philosophical physician, one who duly estimates the importance and dignity of his profession, can be satisfied to confine his observations to one single species from the immense storehouse of living material with which he is surrounded, all equally the objects of the wisdom and beneficence of one Supreme Architect,—curiosity alone, it might be supposed, would impel the investigator to scrutinize the philosophy of organized structure, and the phenomena of life in all the links of the great chain of beings to whom any modification of these have been imparted: of paramount importance is the study of the diseases which afflict the inferior order of animals, particularly of those domestic kinds that so eminently contribute to the comfort and necessities of civilized man. The elevated character of such pursuits has occasionally been duly estimated by the most eminent teachers of medicine;—the immortal Rush, Hunter, Camper, with many other distinguished names, have ably demonstrated the useful results to be obtained from the study of the diseases of the inferior animals. As early as the year 1776, this subject attracted the attention of the French government, when the "Royal Veterinary School" was established at *Alfort*, six miles southeast of Paris. For more than sixty years the great utility of such an institution has been satisfactorily tested; and some of the most eminent physicians have esteemed it an honour to serve as professors, among whom the names of Vicq d'Azyr, Daubenton, Fourcroy, and Desmarest, are prominent. The Cabinet of Comparative Anatomy and Pathology, composed of most extensive and curious specimens, cannot fail to prove instructive and interesting to the general student. My attention was attracted by the skeleton of the horse, as well as specimens prepared for the muscles, of this and other animals, most of which were surmounted by the skeletons, &c., of man or other animals—an arrangement not only calculated to save room, but to place these objects of *comparison* in the strongest light. By a singular *affectation* of some of our "men of science" in Philadelphia, a similar arrangement of the skeletons of man and horse, at the Academy of Natural Sciences, was ridiculed! There is also attached to this great school a library of domestic zoology and veterinary surgery, a botanical garden, hospitals for sick animals, principally for horses, cows, and dogs, a laboratory for chemistry and natural philosophy, a pharmacy, ground for the cultivation of forage and medicinal plants, a school of practical agriculture, and an amphitheatre, where lectures are delivered upon veterinary medicine and rural economy, besides farriers' shops, buildings for experiments, apiary, &c.

There are three hundred pupils admitted, whose expenses for boarding and schooling are only about seventy dollars per annum. The Minister of War has forty pupils in the school, destined for veterinary service in the cavalry.

Sick animals in the vicinity of the hospital are admitted for treatment, on paying fifty cents a day for a horse, twelve cents for a dog, &c. If the owners are poor, the only charge made is for the animals' keep. In case of a distemper or epidemic occurring among the cattle, the students, or even the professors, are sent to treat them. A favourite Asiatic elephant died of some visceral acute inflammation last spring, at the "Jardin des Plantes;" during his illness, he was treated with the same care and skill, by one of the Alfort professors, as if he had been a human being,—and why not? Surely an animal that lives, as far as possible, according to the organic laws, is a far more dignified creature, than a louzy, degraded, vice-worn, drunken biped, who occupies so great a share of the time and attention of the physician.

The students at Alfort appear to be a respectable and studious class of young men, of from sixteen to twenty-five years of age. They occasionally fall a sacrifice to their zeal: only last week, one of them died of the *glanders*, (morve,) contracted by accidental inoculation whilst dissecting a horse dead of this disease, which is incurable in man.

A similar institution exists at Lyons, for the South of France.

There are several medical veterinary establishments in England, and on the Continent. Nothing of the kind has been attempted successfully in *America*, although of all countries, the agricultural interests (to say nothing of the interests of humanity) exact of us peculiar claims!

Allow me now to conduct your readers over the "*Jardin des Plantes*,"—rather an awkward name, to be sure, for an institution which is equally devoted to every department of natural, and most of the physical sciences. It never was originally restricted to the vegetable kingdom, though botany was at first one of its principal objects, and the names of Jussieu, Tournefort, &c., are indissolubly identified with the early history of this science and this school,—whilst Buffon and Cuvier immortalized their own names in immortalizing the productions of animal life. What delightful and cheering associations and reminiscences are here concentrated within the walls of this great and justly celebrated national establishment! An air of science pervades every spot. Seated beneath the wide-extended arms of the lofty and classical "*Tree of Lebanon*," LAURUS *cedrus*, planted more than a century ago by De Jussieu, and close to the tomb of Daubenton, the eye rests upon extensive beds, where flourish trees, plants, herbs, and flowers, from every clime, and of every hue: or, advancing a little towards the river, the observer enjoys an extensive vista of lengthy avenues, shaded by venerable elms and chesnuts, meeting above to form immense Gothic arches. If, perchance, it be early morning, he may see a gentle, spiritual, and modest man, followed by an attentive crowd of pupils—the Professor of Botany, and his class. The garden is expressly arranged for their convenience—every plant being marked with its scientific and common name, and its nativity indicated.

The whole of the northern side of the garden is devoted to the accommodation of living animals, where every arrangement is made to place them as nearly as possible in a state of nature. The elephants, the giraffe, the camels, the tapir, the kangaroo, the horse, and ox kind, enjoy a large and convenient round house, with little gardens attached, for the exercise of each occupant separately.

The monkeys, of every description, lead an enviable and delightful life here. In the winter they are carefully housed, and warmed with a constant fire. During summer, they all meet and frolic away their hours in an immense wire house, with ropes and poles for exercise, with a refreshing fountain in the middle, and every delicacy to eat. The numerous species of the sheep, antelope, and deer kind, together with extensive aviaries, are equally cared for. The lions, tigers, hyenas, bears, wolves, jackalls, &c., occupy separate and safe cages, or deep fossæ. This portion of the garden is tastefully planted with trees and shrubs, where, at night, the sweet voice of "Philomel" has often cheered the solitary rambles of the stranger.

But it is time to enter the interior of the surrounding large and numerous buildings—those great storehouses, in which are concentrated, and carefully preserved, the riches of nature from every quarter of the globe. Passing under a court, on the left or northern side of the garden, you enter a door between two jaw-bones of the whale, some twenty feet in length, into the "salle d'entré," or the ground floor, of the celebrated Cuvierian Museum of *Comparative Anatomy.* The most striking object in this ante-room, is the plaster model of the skull of the newly discovered fossil animal, the "DINOTHERIUM *giganteum*" of Kaup, discovered in 1836, at Eppelshiem, Grand Duchy of Hesse Darmstadt. This animal displays considerable resemblance in its organization to the hippopotamus and tapir, but is of much more gigantic proportions, the skull being four feet long, three feet wide, and nearly two feet high—the length of the lower jaw, following he curvature, and including the tusk, is five feet seven inches, the tusk alone being one foot seven inches, recurved, the points towards the earth. This ante-room includes other models of fossil animals of great interest. The next, or principal room of this suite, contains one hundred and twenty-seven skeletons, including thirty-nine varieties of dogs. But, immediately upon entering, the attention is absorbed in the contemplation of the neat and elegant skeleton of the enormous whalebone whale, (*Balœna mysticetus,*) from the Cape of Good Hope. This is an object of sublimity, from its grandeur; one can scarcely regard the frame-work of this stupendous fabric without feelings of devotion,—not towards the bones, although in the structure and uses of these alone there is enough for our philosophy,—but in the contemplation of the sublime, the soul is naturally elevated to that Creative Power which first called into existence living creatures so perfectly adapted to all the complicated phenomena of life, from the insensible monade of the dew-drop, to the immense leviathan that sports on the billows of the great deep, and laughs at its storms. Nothing can exceed the neatness and accuracy with which these series of immense bony masses are articulated together. The total length of this specimen, in a living state, was about sixty feet. The skeleton is forty-eight feet long; the chest is eight feet broad; the skull is fourteen feet one inch long, and nine feet one inch broad. From the borders of the upper jaw are suspended a series of whalebone laminæ or plates, (fanons,) varying from one to seven feet in length, diverging as they descend towards the lower jaw, or rather beyond it; there are upwards of four hundred in number, presenting their flattened surfaces towards each other, their interior edges being furnished with delicately formed and closely set fringes,—this whole apparatus constituting one of the most complete nets for entrapping the molluscous animals and small fish which form the principal nourishment of these vast gourmands, who must sacrifice thousands of living beings at every mouthful. These whalebone plates are the only representations of teeth with which this species of whale is provided; a double row, or shorter series of these plates, exists on the inner side or base of the larger, and renders the escape of the small animals impossible. Fifteen or twenty men could easily sit around a table within the circumference of the mouth, and yet so small is the opening into the gullet, or œsophagus of this monster, the largest of the existing race, that an infant one year old could not pass between the os hyoides and palate bones, the vertical diameter of which, in the skeleton, does not exceed six inches, and cannot be much more than half that size when clothed with flesh and cartilage in the recent state; hence it is *physically* impossible that *Jonas* could have been *swallowed* by a whale, although many Jonases might have been conveniently stowed away in this whale's mouth. The head of the cacholot, (*Physctor macrocephalus,*) spermaceti whale, is actually and proportionably larger; but this species also feeds on mollusca and crustacea, &c., and is incapable of swallowing a man whole.

Adjoining, and to the right of this grand room on the ground floor, is another devoted principally to the skeletons of the different varieties of the human race, including three Egyptian mummies, in one of which several of the bones have been fractured and skilfully united without the least deformity, which is no slight testimony in favour of surgery in those days, obtained by Geoffroy St. Hilaire in Egypt, and by him presented to the museum. Here is also the skeleton of "Solyman êl Haleby," a young Syrian, who assassinated General Kleber, commander-in-chief of the Egyptian army; the criminal was impaled alive, and had his head burned to the bone, and endured the most cruel tortures for four hours, without uttering a groan.

Close by is the skeleton of a *Guanche* female, an interesting and almost unique remnant of that curious nation, once the original inhabitants of the Island of Teneriffe, whose bravery resisted the Spanish yoke until the last; they were finally exterminated, but never conquered. The skeleton of Pepin, the celebrated Luneville dwarf, is remarkable for its perfect proportions; it is only three feet high. Adjoining is the plaster model of the upper portions of the skeleton of a Roman female, the metallic bracelets and breast-pin still existing "in situ;" it is embedded in stiff, hard clay, and resembles a fossil.

Around the staircase ascending to the upper series of rooms, are suspended a series of hippopotamic skulls, with the skulls of the varieties of oxen brought to the Parisian market. Having ascended this staircase, you enter a cabinet containing Cuvier's collection of skulls, human and comparative, together with the osteology of the hand and foot.

The principal room of the second story consists of a series composed of ten galleries, containing—1st. The comparative anatomy of the osseous system in detail,—the bones, in particular, of the head of the various classes of animals, elegantly arranged, and etiquetted with great labour and talent. 2d. The comparative anatomy of the teeth, both recent and fossil, together with the skeletons of monkeys, bats, and smaller quadrupeds, to the number of three hundred and three. 3d. The same subject continued, with the addition of the skeletons of six hundred and thirty-nine birds, and some of the larger reptiles, such as crocodiles. 4th. Comparative anatomy of the heads of fishes, and of their organs of mastication. 5th. Comparative myology,—preparations preserved in spirits, and models in wax and plaster. 6th. Comparative anatomy of the brain and nervous system, of the eye, of the skin, and its various appendages, of hair, horn, nails, scales, organs of voice in birds, &c., both spirituous and dried preparations. 7th. Comparative splanchnology,—preparations in spirits, and modelled in wax, including the anatomy of the reproductive organs in the chicken and rabbit. 8th. Same subject continued, together with the blood-vessels, the viscera of the larger animals being injected and inflated. 9th. Organs of reproduction; fœtuses of human monsters in great variety, including "Siamese twins" in abundance; the incubation of the chick in ovo, is beautifully illustrated in wax, from the first to the twenty-second day inclusive; also, a most valuable series of molacological specimens, both in spirits, and representations in wax. 10th. The last of the series is devoted to the craniological collection of the late Dr. Gall. This, though not very extensive, is a choice collection of human skulls, of characters distinguished for good or evil propensities, or of great mental endowments, with numerous plaster busts of similar purport.

The cabinet of human anatomy, appertaining to a distinct professorship, is located on the same floor; this is not open to the public. It contains some rare specimens of mummies of the Egyptian, Gaunche, Gaulois, and South American nations; plaster models, coloured after nature, of a Charruas Indian, of the "Hottentot Venus," and of a negro, all of the originals of the latter having lived and died in Paris. There is also a valuable collection of national skulls.

Descending again to the ground floor, there are two extensive rooms containing one hundred and seventeen complete skeletons of the larger quadrupeds, elephants, rhinoceroses, camels, giraffes, tapirs, together with almost every variety of the horse, cow, and deer kind; skulls of the same; also, fossil remains of the mastodon, elephant, and megatherium.

In referring to this great national and liberally endowed institution, justly the pride of the French, I must confine my remarks to this hasty and imperfect sketch of the anatomical department, as more intimately the object of professional interest, and to convince the medical student, who has fairly entered the temple of knowledge, that he will not find himself among the "errores loci," should he make his daily devotions at this shrine. The mere casual observer, if he love nature, cannot fail to be very much interested in visiting the magnificent galleries of zoology, and the splendid new hall, just finished, and devoted to geology, mineralogy, and botany. In the middle of the latter stands a noble statue, in Italian marble, of the "genius loci," the immortal Cuvier, sculptured by David. The renowned author is represented clothed in his robes of state, and decorated with the insignia of civic honours; on his left is a column, surmounted by a globe; on the face of the column is written the titles of those works on which rests principally his claims to pre-eminence, viz., "Leçons d'Anatomie Comparée, Mémoires pour servir à l'histoire et à l'Anatomie des Mollusques, Le Régne Animal distribué d'après son organisation, Discours sur les revolutions du Globe, Recherches sur les ossements fossiles, Rapports sur l'état de l'instruction publique en Hollande, en Allemagne, en Italie, Rapports sur les prix décennaux, Rapport sur les progrés des sciences naturelles, Histoire naturelle des Poissons, Eloges historiques. The left hand of the statue rests on the globe, the forefinger inserted into a large fissure, or rather fracture, in allusion to his researches into the history of the crust of the earth, whilst with the right arm elevated he beckons to his contemporaries to come and observe the curious objects contained beneath.

The professors and administrators of the "Jardin des Plantes" enjoy no sinecures, as such; they are a hard-working, industrious, and zealous class of men, eminently distinguished each in his own peculiar department. Having been long in correspondence with some of them, and honoured with a diploma as correspondent member, on the recommendation of Baron Cuvier, I enjoy peculiar advantages and privileges in my scientific pursuits.

On receipt of the intelligence of the recent unfortunate destruction of my cabinet of comparative anatomy, by fire, the professors testified their most sincere sympathy, considering the disaster a public as well as an individual loss, and unanimously voted to me specimens of the duplicates contained in their invaluable museum. These, together with other curious and beautiful specimens to be gathered in this immense metropolis, will form a rich nucleus for a new collection, which will soon be as numerous, at least, if not so unique, as the original.

Before closing this letter, I ought to refer to the lectures on *animal magnetism* that are at present delivered twice a week in the Athenæum, by Dr. le Baron Duportet, the same gentleman who caused so much discussion last year in London, and who converted Professor Elliotson to his belief in animal magnetism. In company with Dr. Mott, Dr. Knox, and others, I have frequently attended Dr. D.'s lectures, and witnessed his wonderful powers over the mental faculties of many of his class, of all ages and sexes. The effects produced are epileptic, cataleptic, hysterical, convulsive, natural sleep, and somnambulism, in different temperaments. In some few instances no effect whatever followed the best efforts of the operator. He has not attempted clairvoyance. My personal acquaintance with Dr. Duportet is slight, but he enjoys a good reputation, and is considered above voluntary deception; and some of his cases could not by any possibility have been the result of deception or conceit. These effects are highly curious, and bordering on the miraculous, yet not more so than the phenomena of terrestrial magnetism! As relates to the exhibition in question, it is impossible to come to any very satisfactory conclusion, unless all the persons experimented upon were personally known to me; but such as it is, I should certainly have regretted not to have witnessed it.

In closing the present communication, I have to request that you will publish the following list of *gratuitous* lectures delivered in Paris,—by which you will confer a favour on all who may hereafter visit this city in search of scientific instruction.

*Programme of the Courses which commenced April,* 1839, *in the Royal College of France, School of Medicine, School of Pharmacy, Sorbonne, School of Law, Royal Library.*

COLLEGE OF FRANCE.

On the Law of Nature and of Nations—Prof. De Portets.
Arabian—Caussin de Perceval.
History and Moral Philosophy—Michelet.
Persian—Amedée Jaubert.
Mathematics—Lacroix or Libri.
Turkish—Alex. Desgranges.
General and Experimental Natural Philosophy—Savart.
French Literature—J. J. Ampère.
Latin Poetry—Tissot or Dervailly.
Hebrew, Chaldäic, and Syriac—Quatremère.
Latin Eloquence—Burnouf, sen., or Lemaire.
Sanscrit—Eugene Burnouf.

On Astronomy—Prof. Binet.
Political Economy—Rossi.
Greek and Latin Philosophy—Bartholomew Saint-Hilaire.
Natural Philosophy and Mathematics—Biot or Liouville.
Natural History of Inorganic Bodies—Elias de Beaumont.
Archeology—Letronne.
Chinese and Tartar-Mandchou—Stanislas Julien.
Medicine—Magendie.
Greek—Boissonnade.
Natural History of Organised Bodies—Duvernoy.
Chemistry—Thénard or Pelouze.

SCHOOL OF MEDICINE.

On Medical Physics—Prof. Pelletan.
Therapeutics and Materia Medica—Cazenave.
Obstetrics, and Diseases of Women and Children—Moreau.
Hygiene—Royer-Collard.
Pathology and General Therapeutics—F. Dubois.
Surgical Pathology—Marjolin or Gerdy.
Medical Natural History—Richard.
Physiology—Bèrard.
Medical Pathology—Duméril or Andral.
Pathological Anatomy—Cruveilhier.
Pharmacy and Organic Chemistry—Dumas.
Clinical Surgery—Roux, at the Hôtel-Dieu; Jules Cloquet, at the Faculty Hospital; Velpeau at La Charité; Sanson, sen., at La Pitié.
Clinical Medicine—Fouquier and Bouillaud, at La Charité; Chomel at the Hôtel-Dieu.
Clinical Obstetrics—Rostan and Paul Dubois at the Faculty Hospital.

SCHOOL OF PHARMACY.

On Organic Chemistry—Prof. Walter de Clanbry.
Toxicology—Caventou.
Pharmacy—Chevalier.
Pharmaceuto-Vegetable Natural History—Guibourt.
Botany, Organography, and Physiology—Geriart.
Practical and Descriptive Botany—Clarien.

SORBONNE.

On Chemistry—Prof. Balard.
Mechanics—Poisson.
Vegetable Organography—Augustus Saint-Hilaire.
History of Ancient Philosophy—Cousin or Vacherot.
Mineralogy—Beudant.
Ancient History—Lacretelle or Rousseauw Saint-Hilaire.
Practical Mechanics—Poncelet.
Greek Literature—Boissonade or Jules David.
Latin Poetry—Patin.
Vegetable Physiology and Anatomy—Mirbel.
Philosophy—Jouffroy or A. Garnier.
On Anatomy, Comparative Physiology, Zoology—Prof. De Blainville.
Geography—Guigniaut.
Physic—Despretz.
Modern History—Guizot or Lenormant.
Doctrine of Probabilities—Libri.
French Poetry—Saint-Marc Girardin.
Foreign Literature—Fauriel.
Geology—Constant Prévost.
History of Modern Philosophy—Royer-Collard or Damiron.
Differential and Integral Calculus—Lacroix or Lefebvre de Fourcy.
Latin Eloquence—Leclerc or Charpentier.
French Eloquence—Villemain or Géruzez.

SCHOOL OF LAW.

On the Civil Code, (first year)—Prof. Demante and Oudot.
On the Civil Code, (second year)—Bugnet and Valette.
On the Civil Code, (third year)—Duranton and Perreyve.
On French Constitutional Law—Rossi.
Comparison of Penal Legislation—Ortoian.
Criminal Legislation, and Civil and Criminal Suits—Berriat Saint-Prix, and Delzers.
History of Roman and French Law—Poncelet.
Law of Administration—De Gérando.
Law of Nations—Royer-Collard.
Institutes of Justinian, and Roman Law—Blondeau and Ducauroy.
The Pandects—Pellat.
Commercial Code—Bravard.

ROYAL LIBRARY—LIVING ORIENTAL LANGUAGES.

Course of Hindostanese—Prof. Garcin de Tassy.
Common Arabian—Caussin de Perceval.
Armenian—Le Vaillant de Florival.
Persian—Quatremère.
Modern Greek and Greek Paleography—Hase.
Turkish—Amédée Jaubert.
Arabian—Reynaud.

CONSERVATORY OF THE ARTS AND TRADES.

On Agricultural Chemistry—Prof. Marcelin Pouillet.
Geometrical Drawing, Demonstrations, Mechanics—Charles Dupin.
Physics applied to the Arts, with the Demonstration of Machines—Pouillet.
Cultivation—Leclerc-Thouin.
Chemistry applied to the Arts—Clement Désormes.
Hydraulics—L. Moll.
Domestic Economy—Bianqui, sen.
Elementary and Descriptive Geometry—Gaultier.
Drawing of Machines, and Architecture—Armengaud, sen.
Figure and Ornamental Drawing—Dervailly.

*Summer Course of Lectures delivered at the Garden of Plants.*

On Physic—Prof. Beckerel.
Mineralogy—Alex. Brongniart.
Botany—Ad. Brongniart.
Chemistry—Gay Lussac.
Chemistry applied—Chevreuil.
Geology—Cordier.
Zoology—Geoffroy, Mammiferes and Birds; Dumeril, Reptiles and Fishes; Valenciennes, Mollusques and Zoophites; Andouin, Articulated Animals.
Human Anatomy—Serres.
Comparative Anatomy—Blainville.
Physiology—Flourens.
Agriculture—Mirbel.
Practical Botany—Jussieu.

Astronomy, by Prof. Arago, at the Royal Observatory.

Drawing, Painting, Sculpture, &c., at the School of Arts and Design.

Besides numerous private lectures before clubs and societies.

The following notice, to be found in Galignani's Gazette of the 15th inst., is worth recording here:—"M. Hardy, head gardener at the Luxembourg, gives public and gratuitous lectures every Tuesday and Friday mornings, at 8 o'clock, on the grafting and pruning of fruit trees, in the nursery ground of that palace."

